# Plastic-Degrading Potential across the Global Microbiome Correlates with Recent Pollution Trends

**DOI:** 10.1128/mBio.02155-21

**Published:** 2021-10-26

**Authors:** Jan Zrimec, Mariia Kokina, Sara Jonasson, Francisco Zorrilla, Aleksej Zelezniak

**Affiliations:** a Department of Biology and Biological Engineering, Chalmers University of Technology, Gothenburg, Sweden; b Department of Biotechnology and Systems Biology, National Institute of Biology, Ljubljana, Slovenia; c Novo Nordisk Foundation Center for Biosustainability, Technical University of Denmark, Kongens Lyngby, Denmark; d MRC Toxicology Unit, Cambridge, United Kingdom; e Institute of Biotechnology, Life Sciences Center, Vilnius University, Vilnius, Lithuania; Albert Einstein College of Medicine; California Institute of Technology

**Keywords:** bioinformatics, environmental microbiology, metagenomics, microbial ecology, plastic pollution

## Abstract

Biodegradation is a plausible route toward sustainable management of the millions of tons of plastic waste that have accumulated in terrestrial and marine environments. However, the global diversity of plastic-degrading enzymes remains poorly understood. Taking advantage of global environmental DNA sampling projects, here we constructed hidden Markov models from experimentally verified enzymes and mined ocean and soil metagenomes to assess the global potential of microorganisms to degrade plastics. By controlling for false positives using gut microbiome data, we compiled a catalogue of over 30,000 nonredundant enzyme homologues with the potential to degrade 10 different plastic types. While differences between the ocean and soil microbiomes likely reflect the base compositions of these environments, we find that ocean enzyme abundance increases with depth as a response to plastic pollution and not merely taxonomic composition. By obtaining further pollution measurements, we observed that the abundance of the uncovered enzymes in both ocean and soil habitats significantly correlates with marine and country-specific plastic pollution trends. Our study thus uncovers the earth microbiome's potential to degrade plastics, providing evidence of a measurable effect of plastic pollution on the global microbial ecology as well as a useful resource for further applied research.

## INTRODUCTION

The demands for plastic production are increasing annually despite plastic waste pollution presenting a major global environmental problem. The majority of plastic products end up in landfills or dispersed in the environment (1), with inadequate waste management leading to an estimated 9 to 14 million metric tons of plastic entering the ocean every year (2) on top of the already accumulated ∼150 million metric tons (3). Even plastic additives such as phthalate compounds, frequently used as plasticizers, are a major source of concern due to their overuse in a variety of different products and adverse health effects (4, 5). While some thermoplastics (polyethylene [PE], polypropylene [PP], polyethylene terephthalate [PET], polyvinyl chloride [PVC], and phthalic acid [PA]) can be recycled, contaminated and composite plastics as well as thermosets (polyurethane [PU] and vinyl esters) cannot be remolded or heated after the initial forming (6, 7). Although the durability of man-made synthetic plastics facilitates their persistence in the environment, the synthetic polymers, like natural polymers (e.g., polysaccharides), can serve as a microbial carbon source (8–10). Microorganisms thus mediate a number of plastic biodegradation reactions across different environments (11–16), and even plastics such as PET (10) and PU (17) can be transformed and metabolized by microbial species. However, despite this widespread degradation capability, the true microbial potential for plastic degradation across different global habitats is not yet fully understood.

The isolation, identification, and characterization of microorganisms with plastic-degrading potential are frequently conducted from aquatic environments (18–21), waste disposal landfills (22–25), or places that are in direct contact with the plastic, such as plastic refineries (26–28). However, growing microorganisms outside their natural environments using conventional approaches is extremely challenging (29) and limits the amount of isolated species that can be cultured and studied to as little as 1% or lower (30). Studying single microbial isolates also limits our understanding of the microbial ecology of plastic degradation, where microbial consortia have been found to act synergistically, producing more enzymes and degrading plastics more efficiently than individual species (31, 32). Likewise, localized analyses from single locations hinder our understanding of the global environmental impact of plastic materials (33). On the other hand, with advances in environmental DNA sequencing and computational algorithms, metagenomic approaches enable the study of the taxonomic diversity and identification of the functional genetic potential of microbial communities in their natural habitats (33–35). For example, global ocean sampling revealed over 40 million mostly novel nonredundant genes from 35,000 species (35), whereas over 99% of the ∼160 million genes identified in global topsoil cannot be found in any previous microbial gene catalogue (34). This indicates that global microbiomes carry an enormous unexplored functional potential, with unculturable organisms as a source of many novel enzymes (30). Identification of such enzymes involved in the biological breakdown of plastics is an important first step toward a sustainable solution for plastic waste utilization (36, 37). However, despite the availability of experimentally determined protein sequence data on plastic-degrading enzymes (10, 38–43), no large-scale global analysis of the microbial plastic-degrading potential has yet been performed.

In the present study, we explored the global potential of microorganisms to degrade plastics. We compiled a data set of all known plastic-degrading enzymes with sequence-based experimental evidence and construct a library of hidden Markov models (HMMs), which we used to mine global metagenomic data sets covering a diverse collection of oceans, seas, and soil habitats (34, 35, 44, 45). By controlling for false positives using gut microbiome data (46), we compiled a catalogue of over 30,000 nonredundant enzyme homologues with the potential to degrade 10 different plastic types. Comparison of the ocean and soil fractions shows that the uncovered enzymatic potential likely reflects the major differences related to the composition of these two environments. Further analysis of metagenome-assembled genomes in the ocean reveals a significant enrichment of plastic-degrading enzymes within members of the classes *Alphaproteobacteria* and *Gammaproteobacteria* and supports the notion that enzyme abundance increases with sea depth as a response to plastic pollution and not merely taxonomic composition (47–50). By relating the identified enzymes to the respective habitats and measured environmental variables within the soil and ocean environments, we further showed that the abundance of the uncovered enzymes significantly correlates with both marine and country-specific plastic pollution measurements (51–56), suggesting that the earth's microbiome might already be adapting to current global plastic pollution trends.

## RESULTS

### The global microbiome harbors thousands of potential plastic-degrading enzymes.

**FIG 1:**
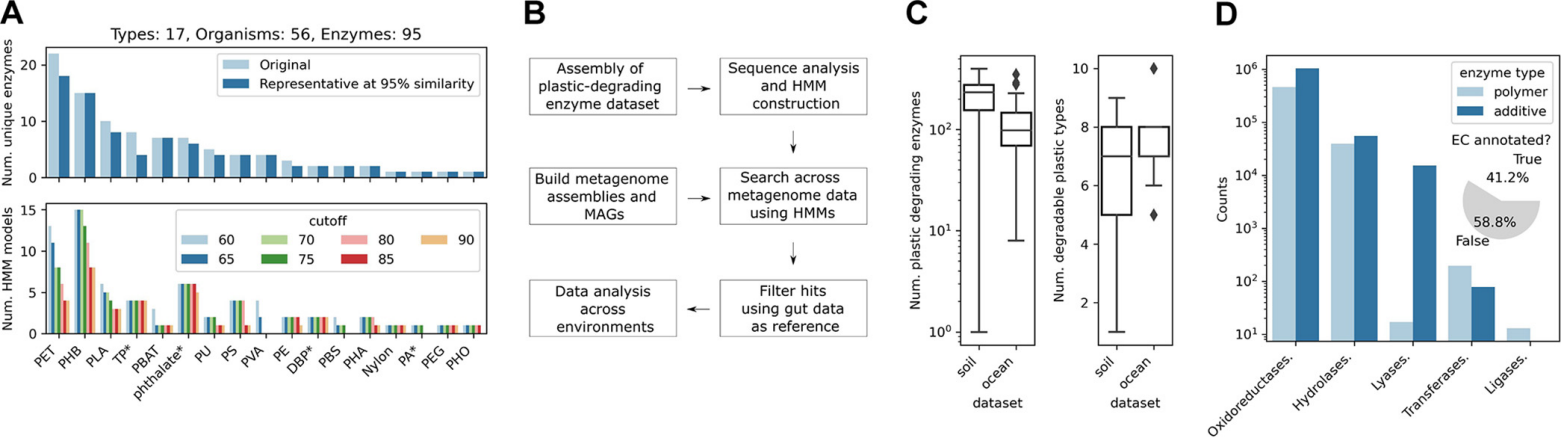
Global microbiome harbors thousands of potential plastic-degrading enzymes. (A) Compiled enzyme data set and representative sequences obtained by clustering (95% sequence identity cutoff), covering the major types of pollutant plastics (PVA, polyvinyl alcohol; PLA, polylactic acid; PU, polyurethane; PHB, polyhydroxybutyrate; PBS, polybutylene succinate; PET, polyethylene terephthalate; PBAT, polybutylene adipate terephthalate; PE, polyethylene; PEG, polyethylene glycol; PHO, poly(3-hydroxyoctanoate)) and additives/plasticizers (phthalate; PA, phthalic acid; DBP, di-*n*-butyl phthalate; TP, terephthalic acid). The lower plot shows the final constructed HMMs across the different sequence identity cutoffs. (B) Schematic overview of the implemented and applied procedures in this study. (C) Number of plastic-degrading enzyme hits and degradable plastic types across the ocean and soil microbiome fractions. (D) Enzyme classes (EC) predicted with orthologous function mapping (66) at the topmost EC level. (Inset) Number of EC annotated results.

To probe the potential for plastic degradation across the global microbiome, we compiled a data set of known enzymes with experimental evidence of plastic-modifying or -degrading activity from published studies (10, 38–42, 57–62) and databases (43), including a total of 95 sequenced plastic enzymes spanning 17 different plastic or additive types from 56 distinct microbial species (Fig. 1A; also, see “Enzyme data set and construction of HMMs” in Materials and Methods). The types of plastics (13 types) and plastic additives (4 types of phthalate-based compounds) (Fig. 1A; additives are marked with asterisks) spanned the main types of globally produced plastics that constitute the major fraction of global plastic waste (1), except for PP and PVC, for which no representatives could be found (Fig. S1A). To enable efficient searching across global metagenomic data sets, we built HMMs (63) by including the known homologous sequences from the UniProt TrEMBL database (64) (Fig. 1B; Fig. S1B and C). Briefly, we clustered the known enzymes to obtain representative sequences (95% sequence identity) (Fig. 1A) and used these to query the UniProt TrEMBL database and obtain an expanded data set of a total of 16,834 homologous enzyme sequences (*E*-value < 1e−10) (Fig. S1C). Each group of enzyme sequences at a given BLAST sequence identity cutoff ranging from 60% (65) to 90% was then clustered (95% sequence identity) to obtain groups of representative sequences that were used to construct a total of 1,201 HMMs (Fig. 1A; Fig. S1; see “Enzyme data set and construction of HMMs” in Materials and Methods).

10.1128/mBio.02155-21.1 FIG S1 Sequence analysis and construction of HMMs. (A) Number of initial experimental enzyme sequences and amount of waste generated in 2010 per plastic type (1). (B) Number of UniProt sequences matching (*E*-value < 1e−10) the representative (95% sequence identity) query sequences (Fig. 1A) at different sequence identity cutoffs from 60% to 90% and used for constructing the HMMs. (C) Total number of UniProt sequences identified (*E*-value < 1e−10) across all representative (95% sequence identity) query sequences at different sequence identity cutoffs and used in the HMMs. (D) Number of unique query sequences at different sequence identity cutoffs in the HMMs. (E) Average number of sequences used per query sequence in the HMMs. (F) Number of HMMs according to the sequence identity cutoff and plastic type. Download FIG S1, JPG file, 1.2 MB. Copyright © 2021 Zrimec et al. 2021 Zrimec et al. https://creativecommons.org/licenses/by/4.0/ This content is distributed under the terms of the Creative Commons Attribution 4.0 International license.

**FIG 2:**
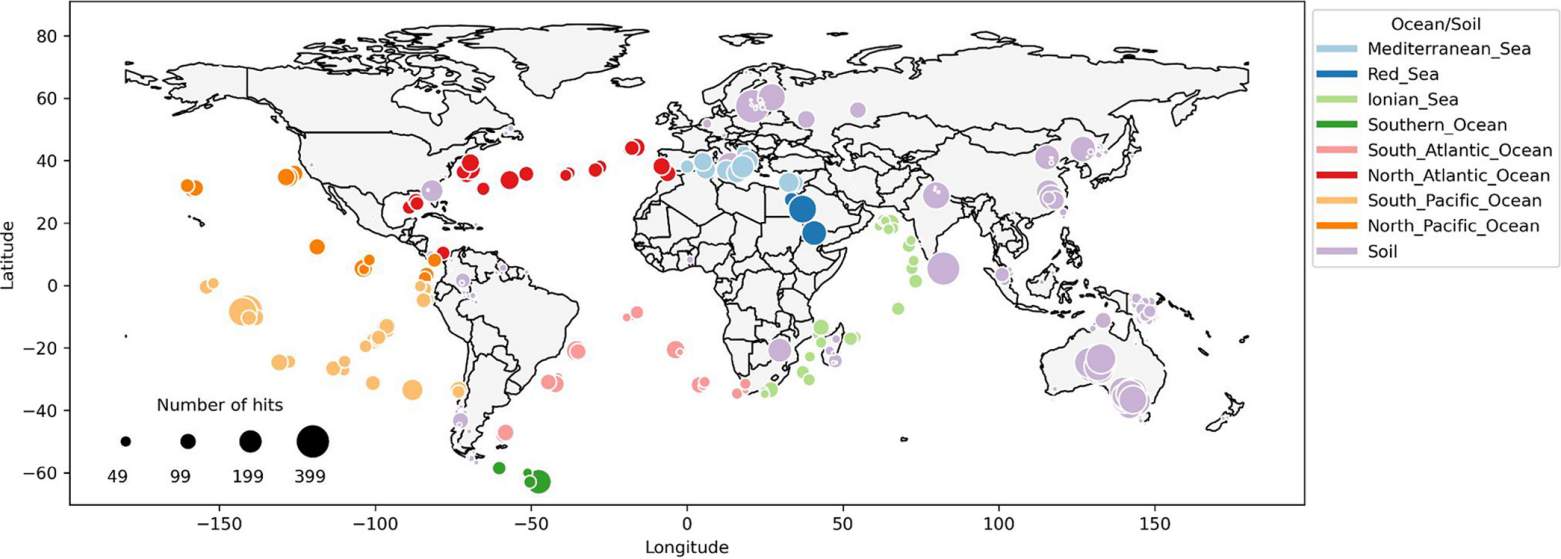
Plastic-degrading enzymes across the global microbiome. Depicted are 11,906 enzyme hits in the ocean and 18,119 in the soil data sets, obtained by constructing HMMs of known plastic-degrading enzymes and querying them across metagenomic sequencing data sets. The potential to degrade up to 10 and 9 different plastic types was observed in the respective ocean and soil fractions (Fig. S3A).

The HMMs were then used to search for homologous sequences from the metagenomes spanning 236 sampling locations (see “HMM queries across metagenomes and data filtering” in Materials and Methods; Fig. 2) that included global ocean (35), global topsoil (34), and additional Australian (45) and Chinese (44) topsoil projects (see “Metagenome assemblies and MAGs” in Materials and Methods; Table 1). With over 73% of orthologous groups shared between gut and ocean microbiomes (35), a high number of false-positive identifications would be expected, as certain enzymes might have related evolutionary ancestry but no plastic degradation activity. Thus, as a control, we filtered the environmental hits by comparing them to those in the gut microbiome (46), where, to our knowledge, no plastic-degrading species have yet been found. Briefly, for each HMM, precision and recall were computed by comparing the corresponding hits in the global microbiomes to those in the gut microbiome; to minimize the risk of false positives, models with hits in the global microbiomes with scores above a precision threshold of 99.99% and an area under the precision-recall curve (AUC) of 75% were retained (Fig. S2; see “HMM queries across metagenomes and data filtering” in Materials and Methods). The final filtered results with the global microbiomes contained 121 unique HMMs, of which 99 matched (*E*-value < 1e−16) ocean samples and 105 matched soil samples, representing 10% of the initial HMMs used prior to filtering (Table 1). Consequently, an average of 1 in 4 organisms in the analyzed global microbiome was found to carry a potential plastic-degrading enzyme (Table 1).

**TABLE 1:** Overview of the metagenomic data sets and results analyzed in the study^*a*^

Data set	No. of:								
	Genes	Samples	HMMs	Hits	Plastic types	Polymers	Additives	Hits per gene	Organisms per hit
soil_Australia (45)	78,849,927	46	99	11,093	9	4,224	6,869	1.41e−04	3.59
soil_global (34)	21,248,672	261	84	6,175	9	1,098	5,077	2.91e−04	1.74
soil_China (44)	6,536,825	6	50	851	7	273	578	1.30e−04	3.88
ocean (35)	107,735,703	139	99	11,906	10	7,232	4,674	1.11e−04	4.57
Weighted avg	53,592,782	113	83	7,506	9	3,207	4,300	1.40e−04	3.61
Total	214,371,127	452	121	30,025	10	12,827	17,198		

a “Hits” and “plastic types” refer to the number of plastic-degrading enzyme hits and number of degradable plastic types, respectively; “polymers” and “additives” specify the amount of plastic polymer and additive-degrading hits, respectively.

10.1128/mBio.02155-21.2 FIG S2 Filtering of hits using gut reference data. (A) Precision-recall curves and (B) area under the receiver operating characteristic (AUC) curve with the 99 HMMs that returned results in the ocean fraction. Download FIG S2, JPG file, 0.3 MB. Copyright © 2021 Zrimec et al. 2021 Zrimec et al. https://creativecommons.org/licenses/by/4.0/ This content is distributed under the terms of the Creative Commons Attribution 4.0 International license.

10.1128/mBio.02155-21.3 FIG S3 Plastic-degrading enzyme hits and degradable plastic types across the ocean and soil fractions. (A) Number of plastic-degrading enzyme hits across different plastic types at ocean and soil sampling sites. (B) Number of degradable plastic types (Num. plast) and number of plastic-degrading enzyme hits (Num. hits) partitioned according to plastic polymer-degrading or additive-degrading function. Download FIG S3, JPG file, 0.4 MB. Copyright © 2021 Zrimec et al. 2021 Zrimec et al. https://creativecommons.org/licenses/by/4.0/ This content is distributed under the terms of the Creative Commons Attribution 4.0 International license.

Altogether, we identified 11,906 enzyme homologues in the ocean and 18,119 in the soil data sets (Fig. 2). Referring to the total number of unique plastic types that can be degraded based on the identified hits, the recovery of the 17 unique plastic types was ∼60%, 10 in the ocean and 9 in the soil data sets. Of these, 38 HMMs matched 43% of hits corresponding to the 6 plastic polymers (Fig. S3A) (polybutylene adipate terephthalate [PBAT], polyethylene glycol [PEG], PET, polyhydroxybutyrate [PHB], polylactic acid [PLA], and PU), and 83 HMMs identified 57% of hits corresponding to the 4 additives (Fig. S3A; di-*n*-butyl phthalate [DBP], PA, terephthalic acid [TP], phthalate). Specifically, of the plastic polymer enzyme hits, PU was found only in the ocean and not in the soil microbiome, whereas >2-fold-larger amounts of PEG, PBAT, and PHB and a 2-fold smaller amount of PET were found in the ocean fraction than the soil fraction (Fig. S3A). The number of hits corresponding to additives was significantly larger (Fisher's exact test one-tailed *p*-value = 5.4e−6) in the soil fraction than the ocean fraction, representing 69% of the total amount of soil hits compared to 39% in the ocean fraction and resulting in an almost 4-fold increase in the average number of additive-degrading hits across the soil sampling sites (Fig. S3B). On the other hand, the overall numbers of polymer-degrading hits across the samples were similar in both the soil and ocean fractions, with a 15% larger number observed in the soil samples (Fig. S3B). The resulting number of all hits, including polymers and additives was thus, on average, over 2-fold larger across the soil samples than in the ocean samples, whereas the numbers of distinct degradable plastic types were equal (Fig. 1C). These results were, however, much more variable across the soil fraction, where, for instance, the variability of the number of hits across soil sampling sites was over 4-fold greater than in the ocean fraction (Fig. 1C).

The identified enzyme hits were annotated using orthologous function mapping (66, 67) (see “Enzyme function and environmental data analysis” in Materials and Methods), which assigned EC enzyme classifications for 41% of the hits (Fig. 1D, inset), with the majority of the annotated enzyme classes corresponding to oxidoreductases, hydrolases, and lyases (Fig. 1D). An over-2-fold larger fraction of additive-degrading hits were annotated compared to the polymer-degrading hits, meaning that, whereas approximately one-half of all the additive-degrading hits were annotated, this was the case with only 29% of the polymer-degrading hits (Fig. S4A). Despite similarities in distributions of the general classes across the ocean and soil fractions (Fig. 1D), 37% fewer hits were annotated with the soil fraction (Fig. S4B). Further analysis showed that, indeed, differences in function were present, with the ocean fraction possessing an 11% larger diversity of enzyme functions than soil (Fig. S4D: 40 versus 36 distinct enzyme types with at least 3 occurrences) and 27% of the enzyme functions differing among the two microbiome fractions. The difference between the additive and polymer-degrading hits was, however, already discernible at the level of general enzyme classes (Fig. S4C). Similarly, in both ocean and soil fractions, an almost 3-fold-larger amount of functional diversity was present with the additive-degrading hits than with the polymer-degrading hits, and only a single function (2%) was shared among the additive- and polymer-degrading groups (Fig. S4E).

10.1128/mBio.02155-21.4 FIG S4 Analysis of plastic-degrading enzyme classes (EC) with orthologous function mapping (66). (A) Number of EC-annotated hits across the plastic polymer-degrading and additive-degrading hits. (B) Percentage of EC-annotated hits across the ocean and soil microbiome data sets and polymer/additive-degrading function. (C) General enzyme classes with >3 occurrences partitioned according to the microbiome dataset and polymer/additive-degrading function. (D and E) Eggnog-predicted enzyme classes at the 4th level with >3 occurrences, partitioned according to (D) ocean or soil data set or to (E) polymer- or additive-degrading function. Download FIG S4, JPG file, 2.2 MB. Copyright © 2021 Zrimec et al. 2021 Zrimec et al. https://creativecommons.org/licenses/by/4.0/ This content is distributed under the terms of the Creative Commons Attribution 4.0 International license.

### Earth microbiome's plastic-degrading potential might already be adapting to global pollution trends.

**FIG 3:**
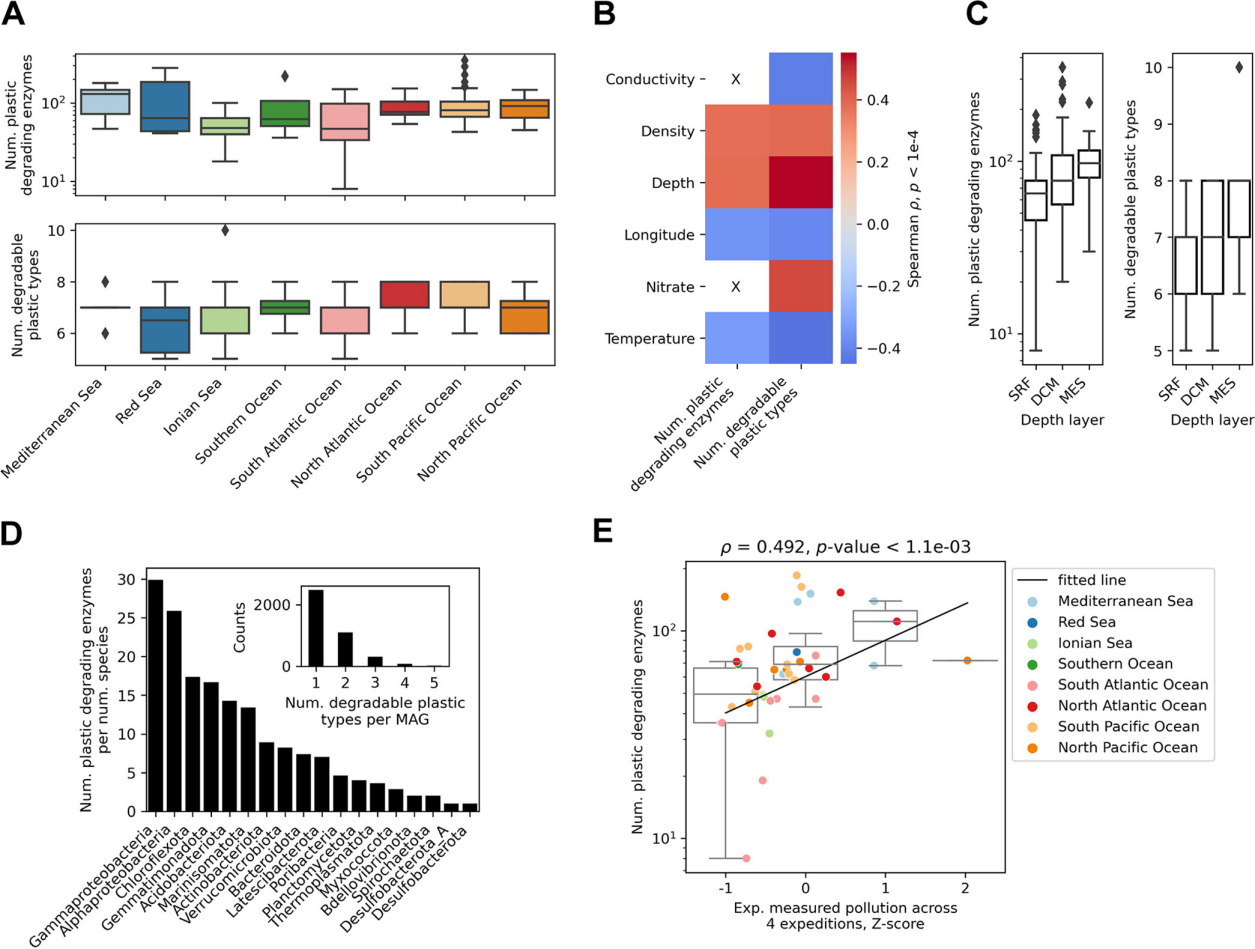
Plastic-degrading potential in the ocean microbiome. (A) Number of plastic-degrading enzyme hits and degradable plastic types found across 8 oceans. (B) Correlation between the number of enzyme hits and degradable plastic types with ocean environmental variables: longitude (°), depth (m), conductivity (mS/cm), temperature (°C), water density (kg/m) and nitrate content (μmol/liter) (35). Only results with a *p*-value of <1e−4 are shown. (C) Number of enzyme hits and degradable plastic types across the ocean sampling depth layers (35). (D) Number of enzyme hits relative to the number of species obtained with the metagenome-assembled genome (MAG) analysis at the phylum level (class level for *Proteobacteria*) (see “Metagenome assemblies and MAGs” in Materials and Methods). (Inset) Number of degradable plastic types per MAG. (E) Correlation of ocean plastic-degrading enzyme hits with experimentally measured plastic pollution across 4 ocean expeditions (51–55) (see “Enzyme function and environmental data analysis” in Materials and Methods). The black line denotes the repeated median fit (122).

The analyzed ocean microbiome spanned 67 locations sampled at 3 depth layers and across 8 oceans (Fig. 2; see “Enzyme function and environmental data analysis” in Materials and Methods). A significant (rank sum test *p*-value < 2.9e−2) increase of plastic-degrading enzyme hits was identified in samples obtained from the Mediterranean Sea and South Pacific Ocean compared to the other locations (Fig. 3A; Table S1), which might reflect the relatively high plastic pollution in these areas (53, 68). A larger amount of pollution in sampling areas in the lower longitudinal region, however, might be indicated by the significant negative correlation (Spearman ρ = 0.393 and 0.357; *p*-value < 1.6e−5) of the numbers of both degradable plastic types and enzyme hits, respectively, with longitude (Fig. 3B; Fig. S5A). Whereas the majority of plastic polymer and additive types were found across all oceans, PU was present only in the Ionian Sea and South Pacific Ocean and PLA was present only in the Ionian Sea, likely reflecting their overall 6-fold-lower content than the other degradable plastic types (Fig. S6A).

10.1128/mBio.02155-21.5 FIG S5 Correlation analysis between (A) ocean microbiome plastic-degrading enzyme hits and environmental variables, (B) the first principal coordinate of PCoA analysis (explaining 25% of the data variance) and environmental variables with the ocean microbiome, (C) soil microbiome plastic-degrading hits and environmental variables, and (D) the first principal coordinate of PCoA analysis (explaining 14% of the data variance) and environmental variables with the soil microbiome. “P. evapotrans.” denotes potential evapotranspiration; NPP is net primary productivity. Download FIG S5, JPG file, 2.6 MB. Copyright © 2021 Zrimec et al. 2021 Zrimec et al. https://creativecommons.org/licenses/by/4.0/ This content is distributed under the terms of the Creative Commons Attribution 4.0 International license.

10.1128/mBio.02155-21.6 FIG S6 Uncovered degradable plastic types across the (A) ocean and (B) soil habitats. “Num. hits” denotes the number of plastic-degrading enzymes found. (C) Sampling locations of plastic waste pollution from 4 ocean expeditions (51–55) that corresponded to the ocean microbiome sampling locations, for which plastic-degrading enzyme hits were identified, at a distance radius of <400 km per sampling site (see “Analysis of global plastic pollution data” in Materials and Methods). (D) Soil sampling locations for which plastic-degrading enzyme hits were identified and which corresponded to the respective country-specific data on inadequately managed plastic waste (56, 71) across 38 countries. Download FIG S6, JPG file, 1.5 MB. Copyright © 2021 Zrimec et al. 2021 Zrimec et al. https://creativecommons.org/licenses/by/4.0/ This content is distributed under the terms of the Creative Commons Attribution 4.0 International license.

10.1128/mBio.02155-21.8 TABLE S1 Rank sum test with the number of plastic-degrading enzyme hits at a specific ocean region or soil environmental habitat versus the other regions or habitats. Download Table S1, TXT file, 0.001 MB. Copyright © 2021 Zrimec et al. 2021 Zrimec et al. https://creativecommons.org/licenses/by/4.0/ This content is distributed under the terms of the Creative Commons Attribution 4.0 International license.

As expected according to published results showing an increasing amount of taxonomic and functional richness with depth (35), we observed measurable depth stratification of the enzyme hits in the ocean samples (Fig. 3C; see “Enzyme function and environmental data analysis” in Materials and Methods). Both the amount of degradable plastic types and enzyme hits were positively correlated with depth (Spearman ρ = 0.552 and 0.384, respectively; *p*-value < 4.3e−6) as well as negatively correlated with temperature (Spearman ρ = 0.451 and 0.336, respectively; *p*-value < 6.7e−5) (Fig. 3B and C; Fig. S5A). This was also supported by principal-coordinate analysis (PCoA) on enzyme hits across samples (see “Enzyme function and environmental data analysis” in Materials and Methods), where the first principal coordinate carrying 25% of the data variance correlated significantly (Spearman ρ = 0.453 and −0.420; *p*-value < 4e−7) with both depth and temperature, respectively (Fig. S5B). We therefore next reconstructed metagenome-assembled genomes (MAGs) in the ocean samples and predicted their taxonomies (see “Metagenome assemblies and MAGs” in Materials and Methods). The results corroborated a significant correlation (Spearman ρ = 0.392 and 0.548; *p*-value < 2.5e−6) between the numbers of degradable plastic types and enzyme hits, respectively, with the number of unique organisms at the family level (Fig. S7A and B; similar results were obtained with other taxonomic levels). We found that, although the majority (62%) of organisms (MAGs) were associated with a single plastic type, 2.5% of them carried enzymes corresponding to 4 or more different plastic types (Fig. 3D, inset; Fig. S7C and D). Analysis of the plastic distribution across species showed that the number of enzyme hits was significantly enriched (Fisher's exact test one-tailed *p*-value < 1.4e−05) within *Alphaproteobacteria* and *Gammaproteobacteria*, which can be expected, since *Proteobacteria* is the most abundant and diverse phylum in the data set (Fig. 3D; Table S2). Nevertheless, the results suggested that the observed plastic-degrading enzyme abundance (Fig. 3D) might be a reflection not merely of taxonomic and functional richness but also of recently uncovered large amounts of plastic pollution below the ocean surface (47–50).

10.1128/mBio.02155-21.7 FIG S7 Analysis of metagenome-assembled genomes. (A and B) Correlation analysis between unique organisms at the family level and (A) number of degradable plastic types or (B) number of plastic-degrading enzyme hits. (C and D) Phylum-level (class-level for *Proteobacteria*) depiction of (C) the number of degradable plastic types and (D) plastic type distribution in the ocean metagenome-assembled genomes. Download FIG S7, JPG file, 1.0 MB. Copyright © 2021 Zrimec et al. 2021 Zrimec et al. https://creativecommons.org/licenses/by/4.0/ This content is distributed under the terms of the Creative Commons Attribution 4.0 International license.

10.1128/mBio.02155-21.9 TABLE S2 Phylum-level (class-level for *Proteobacteria*) enrichment analysis of plastic-degrading enzyme hits in the ocean metagenome-assembled genomes. Download Table S2, TXT file, 0.001 MB. Copyright © 2021 Zrimec et al. 2021 Zrimec et al. https://creativecommons.org/licenses/by/4.0/ This content is distributed under the terms of the Creative Commons Attribution 4.0 International license.

**FIG 4:**
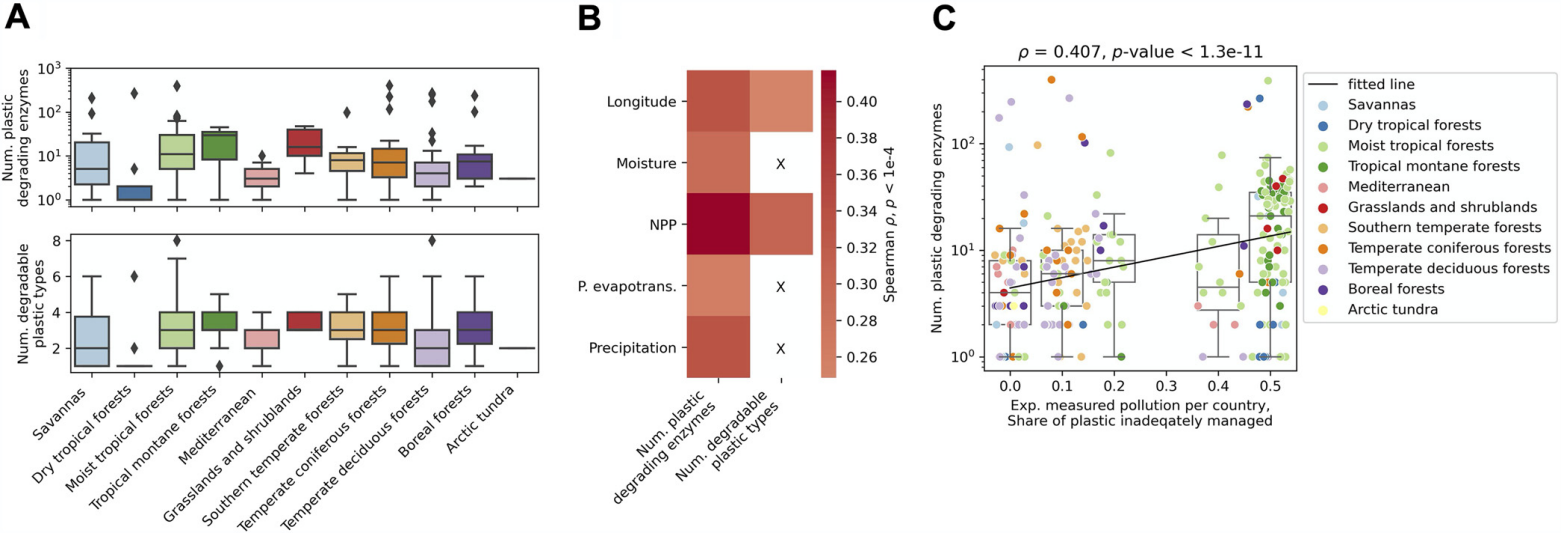
Plastic-degrading potential in the soil microbiome. (A) Number of plastic-degrading enzyme hits and degradable plastic types found across 11 soil habitats. (B) Correlation between the number of enzyme hits and degradable plastic types with soil environmental variables: longitude (°), average monthly moisture content (%), net primary productivity (NPP) (g cm^−2^ year^−1^), and average yearly potential evapotranspiration and precipitation (liters/m^2^) (34). Only results with a *p*-value of <1e−4 are shown. (C) Correlation of soil plastic-degrading enzyme hits with the share of inadequately managed plastic per country (56). The black line denotes the repeated median fit (122).

The analyzed soil microbiome spanned 169 sampling locations across 38 countries and 11 distinct environmental habitats (Fig. 2; see “Enzyme function and environmental data analysis” in Materials and Methods). To ensure the accuracy of cross-habitat and cross-country comparisons, due to the different technical specifications of sample acquisition and processing across the metagenomes (34, 44, 45), here we focused on the uniformly processed global topsoil data set (34), which also represented the largest fraction of the data (163 sampling locations) covering all given countries and habitats. A significant (rank sum test *p*-value < 4.8e−3) increase of plastic-degrading enzyme hits was identified in samples from the moist tropical forest and tropical montane forest habitats compared to the other habitats (Fig. 4A; Table S1). This was corroborated by a significant correlation (Spearman ρ was 0.248 and 0.332; *p*-value < 5e−5) of the numbers of both degradable plastic types and enzyme hits, respectively, with longitude as well as the number of enzyme hits with both the measured annual moisture content (Spearman ρ = 0.292; *p*-value = 6.8e−6) and precipitation levels (Spearman ρ = 0.330; *p*-value  = 4.6e−8) (Fig. 4B; Fig. S5C and D). Interestingly, the soil habitats contained the most distinct differences of plastic content compared to the ocean microbiome, with all degradable plastic types present only in the moist tropical forests and temperate deciduous forests (Fig. S6B). Besides these two areas, PET, for example, was additionally found only in the Mediterranean habitat (Fig. S6B).

Since the results suggested that the plastic-degrading enzyme hits might reflect actual global pollution trends (Fig. 3A and 4A), and considering that global pollution with plastics and microplastics has been an ongoing and steadily increasing problem for over 5 decades (69, 70), we next determined if the global potential for plastic degradation reflected measured plastic pollution in the environment. We obtained data from 4 ocean expeditions surveying plastic pollution across different oceans (51–55) and pooled it to cover 61% of the ocean sampling locations at the surface depth layer from all 8 oceans by matching the closest data points to those of the ocean sampling locations at a maximum radius of 400 km (see the sensitivity analysis in Table S3; see “Analysis of global plastic pollution data” in Materials and Methods; Fig. S6C). Similarly, by obtaining a data set of inadequately managed plastic waste across different countries (56, 71), we achieved 72% coverage of the soil samples across 35 countries (Fig. S6D). Using these common pollution data sets, we indeed observed significant correlation (Spearman ρ = 0.492 and 0.407; *p*-value  < 1.1e−3) between the number of identified enzymes and pollution trends within both the ocean and soil microbiomes, respectively (Fig. 3E and 4C). Strikingly, this observed correlation between the abundance of plastic-degrading enzymes with global pollution suggests that the global microbiome might already be adapting to the effects of global plastic pollution.

10.1128/mBio.02155-21.10 TABLE S3 Sensitivity analysis of plastic-degrading enzyme hits across ocean samples with experimentally measured pollution data (51–55) by increasing the distance radius between the sampling sites. Download Table S3, TXT file, 0.001 MB. Copyright © 2021 Zrimec et al. 2021 Zrimec et al. https://creativecommons.org/licenses/by/4.0/ This content is distributed under the terms of the Creative Commons Attribution 4.0 International license.

## DISCUSSION

Here, we catalogued potential plastic-degrading enzymes, including the majority of massively produced and globally polluting polymers (Fig. 1A; Fig. S1A) as well as the major additives involved in plastic production, identified from metagenomes sampled from soils and oceans across the globe (34, 35, 44, 45) (Fig. 2). We used an initial set of 95 experimentally verified published sequences and expanded it with UniProt sequences to build enzyme sequence models (hidden Markov models [63]) for mining metagenomic data (Fig. 1A and B). We identified a total of 30,000 enzyme hits in the ocean and soil microbiomes (Fig. 2) (11,906 and 18,119, respectively) corresponding to 10 major plastics types, including 6 polymers and 4 additives (Fig. 1C; Fig. S3). Nearly 60% of identified plastic-degrading enzymes did not map to any known enzyme classes (Fig. 1D), suggesting that novel plastic-degrading functional content was uncovered, which is not surprising considering the vast numbers of novel functions uncovered in recent large-scale metagenomic studies (33–35, 50).

To minimize the number of false-positive hits, we used the gut microbiome (46) as a negative control (Fig. 1B; Fig. S2); that is, we assumed that this microbiome is not evolved to degrade plastics, and thus, enzyme hits that are similar to the ones found in the human gut would indicate false positives. Recent studies show that humans might ingest large amounts of plastic particles, such as micro- and nanoplastics (72), as it has recently been discovered that apart from being used in cosmetic products (73), these small particles also enter the food chain and contaminate different types of food and drink (72, 74, 75). Plastic contaminants can have multiple adverse effects on the gut microbiome, according to studies of animal models (74, 76), including impairments in oxidative and inflammatory intestinal balance (77, 78), disruption of epithelial permeability (78, 79), and dysbiosis, where the symbiosis between host and the natural community and abundance pattern of the gut microbiota is disrupted (80, 81). Certain species, such as larvae of Plodia interpunctella (waxworms), *Tenebrio* molitor (mealworms), and Galleria mellonella, were even found to have developed a flora that can degrade polyethylene (82, 83), polystyrene (84, 85), or both plastic types simultaneously (86). However, these organisms might have a highly adapted and specialized microbiome due to their direct exposure and breeding in specific plastic-contaminated habitats (82, 84), whereas, to our knowledge, there have been no documented cases of the human gut microbiome displaying plastic-degrading properties. Therefore, as the procedure of using the human gut microbiome (46) to control for false-positive hits was highly stringent, thus reducing false-positive results at the cost of potentially losing some true hits, we presumed that this was a robust solution to ensure the validity of our findings.

A potential reason for the observed functional differences between the soil and ocean microbiomes (Fig. 1C and D; Fig. S3 and S4) could arise not only from the different plastic availability and pollution trends across these environments (51–53, 56) but also from the general mechanical and chemical differences between these two environments (87). For instance, the ocean is a highly dynamic environment due to its compositional medium with a large degree of mixing. As such, compared to soil, which is in large part composed of solids, one can expect an intrinsically lower community and functional stratification per unit volume in the ocean (35). The increased variability of enzyme hits and degradable plastic types across soil habitats (Fig. 1C; Fig. S7C and D), for instance, was likely a reflection of such differences. Furthermore, the large fluctuations in temperature, salinity, and mechanical forces in the ocean lead to it intrinsically possessing many polymer-degrading properties (88–90), differing from those in the soil (87) and possibly resulting in further preferences in the specific functional content. On the other hand, the soil generally contains a higher observed overall species richness (34, 91), and thus it is likely that certain enzyme families are overrepresented in each environment. This, as well as the fact that plastic additives are likely easier to degrade than the general plastic polymers due to being simpler molecules, could be the reason behind the observed large differences in the additive- versus polymer-degrading content between the ocean and soil fractions (Fig. S3 and S4). The uncovered additive-degrading enzymes in soil also likely corresponded to overrepresented but unknown enzyme classes in soil that could not be identified using the orthogonal mapping procedure (66, 67) (Fig. S4B). Moreover, a potentially larger amount of additive pollution on land than in the oceans might occur due to the majority of industrial activities related to production, disposal, and recycling of plastics being performed on land (92). Here, a frequent and documented problem is the early release (leaching) or migration of plastic additives, such as plasticizers, which include the phthalate compounds analyzed here (92, 93) (Fig. 1A). Plasticizers are most commonly used for improving the mechanical properties of polymeric films and, since they are not necessarily bound to the plastic, they are much easier to release during the use or recycling of the plastic product (92). Such migration has been shown in multiple cases for phthalate-based plasticizers (94–96), possibly leading to their contamination of different food products and the environment (97–99). These additives can potentially also be released by the application of various recycling techniques, especially in underdeveloped countries where the sorting, reprocessing, and recycling conditions are usually uncontrolled (92).

Plastics have been increasingly mass produced ever since the economic and social explosion after the second world war, with the first signs of global plastic pollution concern arising over half a century ago (69, 70), giving ample evolutionary time for microbial functional adaptation to these compounds (50, 100, 101). Such adaptation was recently uncovered with PET-degrading enzymes across ocean metagenomes of planktonic communities (50), where multiple fully functional enzyme variants were found to be evolved from ancestral enzymes degrading polycyclic aromatic hydrocarbons, suggesting that the current PET exposure already provides sufficiently strong selective pressures to direct the evolution and repurposing of such enzymes. Similarly, enzymes degrading other plastic types have been shown to be widely occurring, with numerous homologs in diverse organisms, and likely arose from well-conserved general enzyme classes (102, 103). Indeed, here we found multiple lines of evidence supporting the idea that the global microbiome's plastic-degrading potential reflects recent measurements of environmental plastic pollution. First, we find that taxonomic and functional richness is likely not the only driver of the observed depth stratification of enzyme hits (Fig. 3C). The organisms found to carry the largest numbers of plastic-degrading enzymes (Fig. 3D) do not completely reflect initial taxonomic estimates in the ocean (35), indicating that the plastic-degrading potential also reflects the recently uncovered trends of an increasing amount of plastic pollution below the surface (<200 m) (47), with considerable microplastic pollution in the mesopelagic zone (48), which are potentially stronger drivers of the observed depth stratification (50). Second, certain habitats containing the largest numbers of observed enzyme hits, such as the Mediterranean Sea and South Pacific Ocean (Fig. 3A), are known to be highly polluted areas (53, 68). Last, this prompted us to verify and uncover the significant measurable correlation of both ocean and soil enzyme hits with experimentally measured pollution across oceans and countries from multiple data sets (51–56) (Fig. 3E and 4C), suggesting that the earth microbiome's potential for plastic degradation is already evolving as a response to the rise in environmental pollution.

Considering that natural plastic degradation processes are very slow (e.g., the predicted life span of a PET bottle under ambient conditions ranges from 16 to 48 years [104]), the utilization of synthetic biology approaches to enhance current plastic degradation processes is crucial (105, 106). Moreover, although there is still unexplored diversity in microbial communities, synergistic degradation of plastics by microorganisms holds great potential to revolutionize the management of global plastic waste (36, 37). To this end, the methods and data on novel plastic-degrading enzymes can help researchers by (i) providing further information about the taxonomic diversity of such enzymes as well as understanding of the mechanisms and steps involved in the biological breakdown of plastics, (ii) pointing toward the areas with increased availability of novel enzymes, and (iii) providing a basis for further application in industrial plastic waste biodegradation. As a future perspective, more experimental data on plastic-degrading enzymes is required, with better coverage that more accurately reflects the abundance of plastic types in global waste (Fig. S1A). Improved enzyme coverage can increase the accuracy of computational results in microbial communities and uncovered distributions of plastic-degrading enzymes across different plastic types, as well as possibly enable the study of plastic degradation pathways with multiple enzymes (90).

## MATERIALS AND METHODS

### Enzyme data set and construction of HMMs.

As initial query enzyme data, which could be used to construct hidden Markov models (HMMs) for searching across global microbiomes (Fig. S1B), we compiled a data set of 95 sequenced plastic enzymes spanning 17 plastic types with experimentally observed evidence of plastic modifying or degrading activity from published studies (10, 38–42, 57–62) and databases (43) (Data Set S1 in GitHub repository; see “Software and data” in Materials and Methods). The enzyme data set comprised 13 types of plastics and 4 types of phthalate-based plastic additives (5) (Fig. 1A). To construct the HMMs, representative sequences were first obtained from the above initial data set of enzyme sequences by clustering them using CD-HIT v4.8.1 (107, 108) with default settings, with the exceptions of using a word size of 5, cluster size of 5, and sequence identity cutoff of 95%. To expand the sequence space for building the HMMs, the UniProt TrEMBL database (64) was queried with the representative enzyme sequences using BLAST+ v2.6 (109) with default settings, except for an *E*-value cutoff of 1e−10. For each group of enzyme sequences at a given BLAST sequence identity cutoff ranging from 60% to 90% in increments of 5%, representative sequences were obtained by clustering using CD-HIT with the same parameters as above. Finally, HMMs were constructed using the HMMER v3.3 hmmbuild utility (110) (http://hmmer.org/) with default settings.

### Metagenome assemblies and MAGs.

To construct metagenomic assemblies and metagenome-assembled genomes (MAGs) that could be queried with the HMMs, metagenomic sequencing data were obtained from the Tara Oceans expedition (35), from global (44), Australian (45), and Chinese topsoil projects (34), and from a gut microbiome study (46). From the sequencing data, metagenomic assemblies were reconstructed using MEGAHIT v1.2.9 (111) with the –presets meta-sensitive parameter, except with Tara Oceans data, where the published assemblies were used (35). MAGs were constructed for the ocean data set by first cross-mapping paired-end reads to assemblies with kallisto v0.46.1 (112) to obtain contig coverage information across samples. This information was then input to CONCOCT v1.1.0 (113) to generate a draft bin set. MetaBAT2 v.2.12.1 (114) and MaxBin2 v2.2.5 (115) were also used to generate additional draft bin sets. Finally, the three bin sets were dereplicated and reassembled using metaWRAP v1.2.3 (116) with the parameters -x 10 -c 50 to obtain the final set of MAGs (117). Default settings were used except where otherwise stated.

### HMM queries across metagenomes and data filtering.

For identifying homologous sequences in metagenomes using the constructed HMMs, hmmsearch from HMMER v3.3 (110) was used with default settings. Furthermore, to minimize the risk of false-positive results, we filtered the environmental hits by comparing their bit score to those obtained with the gut microbiome. For each HMM, precision, recall, and the area under the precision-recall curve (AUC) were computed by comparing the corresponding hits in the global microbiomes to those in the gut microbiome. Only models with a minimum of 20 data points and hits in the global microbiomes with an *E*-value cutoff below 1e−16 and scores above a precision threshold of 99.99% and AUC of 75% were retained. Additionally, only the lowest *E*-value and bit score hit were retained for each gene in the global metagenomes. Consequently, the HMM queries across metagenomes identified a total of 30,025 homologous plastic-degrading enzyme hits nonredundant at the amino acid level, comprising 11,906 hits in the ocean and 18,119 in the soil data set (Table 1; see Data Set S2 in the GitHub repository; see “Software and data” in Materials and Methods). The precision-recall analysis was performed using Scikit-learn v0.23.1 (118) with default settings.

### Enzyme function and environmental data analysis.

To annotate the identified plastic-degrading enzyme hits with EC enzyme classifications, we performed orthologous function mapping using Eggnog-mapper v2 (66, 67) with default settings. This led to 41% of the enzyme hits being functionally annotated and used to analyze and compare enzyme functions within and between the microbiome fractions.

Environmental data for the ocean and soil microbiomes were obtained from the supplementary information attached to the respective Tara Oceans (35) and global topsoil (34) publications, with additional data obtained as follows: (i) Tara Oceans data from the PANGEA database (www.pangaea.de) (35) and (ii) global topsoil data from the Atlas of the Biosphere (https://nelson.wisc.edu/sage/data-and-models/atlas/maps.php), with the exception of temperature and precipitation data, which were obtained from the WorldClim database (https://www.worldclim.org/) (34). For the ocean microbiome analysis, the prokaryote-enriched fraction of the ocean data was used by filtering for size-fractionated samples targeting organisms between 0.22 and 3 μm (35). The depth layers at which the ocean microbiome was sampled included (i) the surface water layer (SRF; mean ± standard deviation [SD] of 5 ± 0 m, 63 samples), (ii) the deep chlorophyll maximum (DCM; 71 ± 41 m, 46 samples) layer, and (iii) the mesopelagic zone (MES; 600 ± 220 m, 30 samples) (35). The soil microbiome analysis was performed with the global topsoil data set (34) (Table 1) that incorporated the corresponding environmental data unified across all samples, with the 11 environmental habitats used as defined by Bahram et al. (34): arctic tundra (1 sample), boreal forests (14 samples), dry tropical forests (9 samples), grasslands and shrublands (5 samples), Mediterranean (13 samples), moist tropical forests (88 samples), savannas (14 samples), southern temperate forests (23 samples), temperate coniferous forests (18 samples), temperate deciduous forests (42 samples), and tropical montane forests (34 samples).

For statistical hypothesis testing, SciPy v1.1.0 (119) was used with default settings. All statistical tests were two tailed unless stated otherwise. For correlation analysis, the Spearman correlation coefficient was used. To explore the overall variability in the composition of identified enzyme hits, principal-coordinate analysis (PCoA) was performed using Scikit-bio v0.5.5 (http://scikit-bio.org/) with default settings and the Bray-Curtis distance.

### Analysis of global plastic pollution data.

To analyze if the identified plastic-degrading enzyme hits reflect current global plastic pollution trends, we obtained experimentally measured ocean and country-specific pollution data. For the ocean pollution analysis, data were obtained from 4 published ocean expedition measurements (51–55) either from the supplementary information attached to each publication or from the authors upon email request (54, 55). Since the data were obtained at different specific ocean regions and, on average, corresponded to merely ∼10 microbiome sampling locations per pollution data set, we constructed a single combined data set of pollution measurements that could facilitate the correlation analysis. For this, the data were pooled by standardizing the values using the Box-Cox transform (120) and computing z-scores. With the ocean microbiome sampling locations with identified enzyme hits, only sampling locations corresponding to the surface layer were used, as the pollution measurements were also performed at the ocean surface layer. To determine the optimal distance cutoff, based on which the plastic pollution data were assigned to each microbiome sampling location with identified enzyme hits, a sensitivity analysis was performed at different distance cutoffs, with a radius of 400 km being identified as optimal (Table S3). At each distance cutoff, only the closest pollution measurement point was retained for each sampling location. Similarly, for the soil pollution analysis, published pollution data on inadequately managed plastic waste across different countries (56) were obtained from an online data repository (71) and were standardized using the Box-Cox transform and by computing z-scores. The data on the amount of waste generated in 2010 per plastic type (1) was obtained from the authors upon email request. The Spearman correlation coefficient was used for the correlation analysis between the numerical variables, with numerical range-specific box plots in Fig. 3E and 4C shown to aid data visualization.

### Software and data.

Snakemake v5.10.0 (121), Python v3.6 (www.python.org), and R v3.6 (www.r-project.org) were used for computations. Code for the data analysis and supplementary data sets are available at https://github.com/JanZrimec/Plastic_degrading_microbiome, with additional data to reproduce the analysis published at https://doi.org/10.5281/zenodo.5112372.
