# Supplementary figures and images for: Plastic-Degrading Potential across the Global Microbiome Correlates with Recent Pollution Trends
Source: mBio. 2021 Oct 26;12(5):e02155-21. doi: 10.1128/mBio.02155-21 (PMC8546865; doi:10.1128/mBio.02155-21)

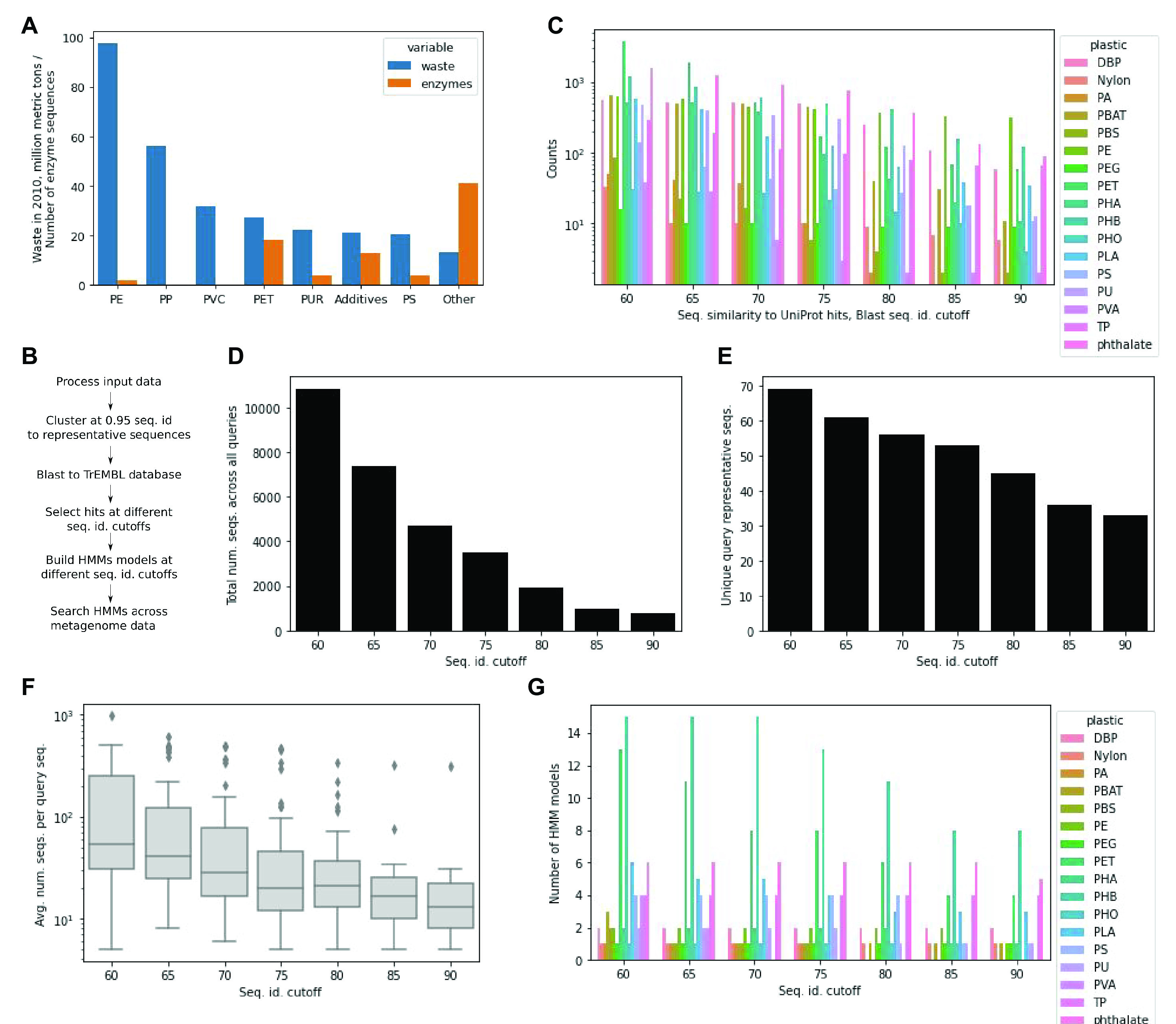

Supplement: FIG S1 [file mbio.02155-21-sf001.jpg]

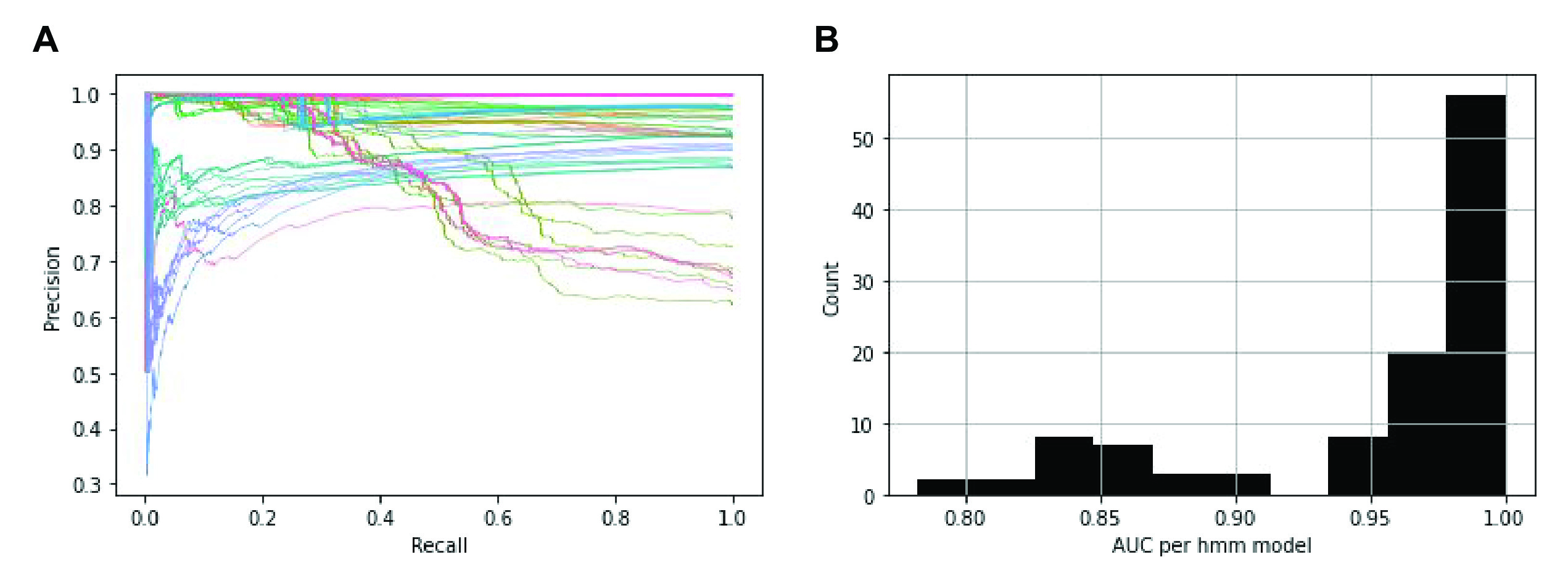

Supplement: FIG S2 [file mbio.02155-21-sf002.jpg]

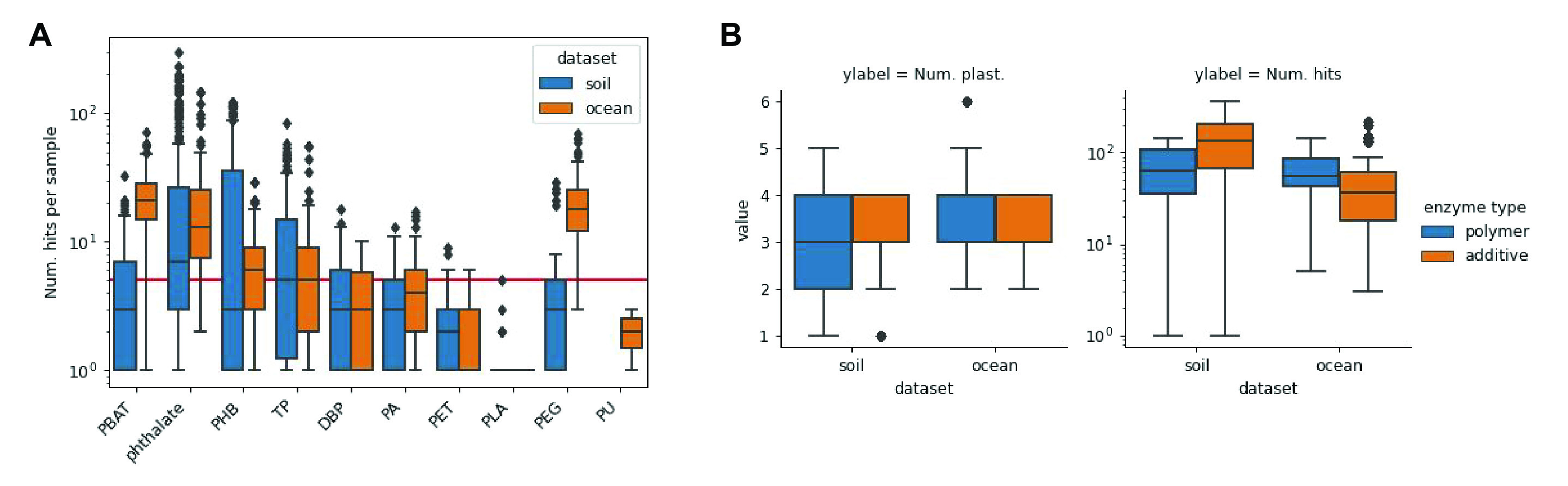

Supplement: FIG S3 [file mbio.02155-21-sf003.jpg]

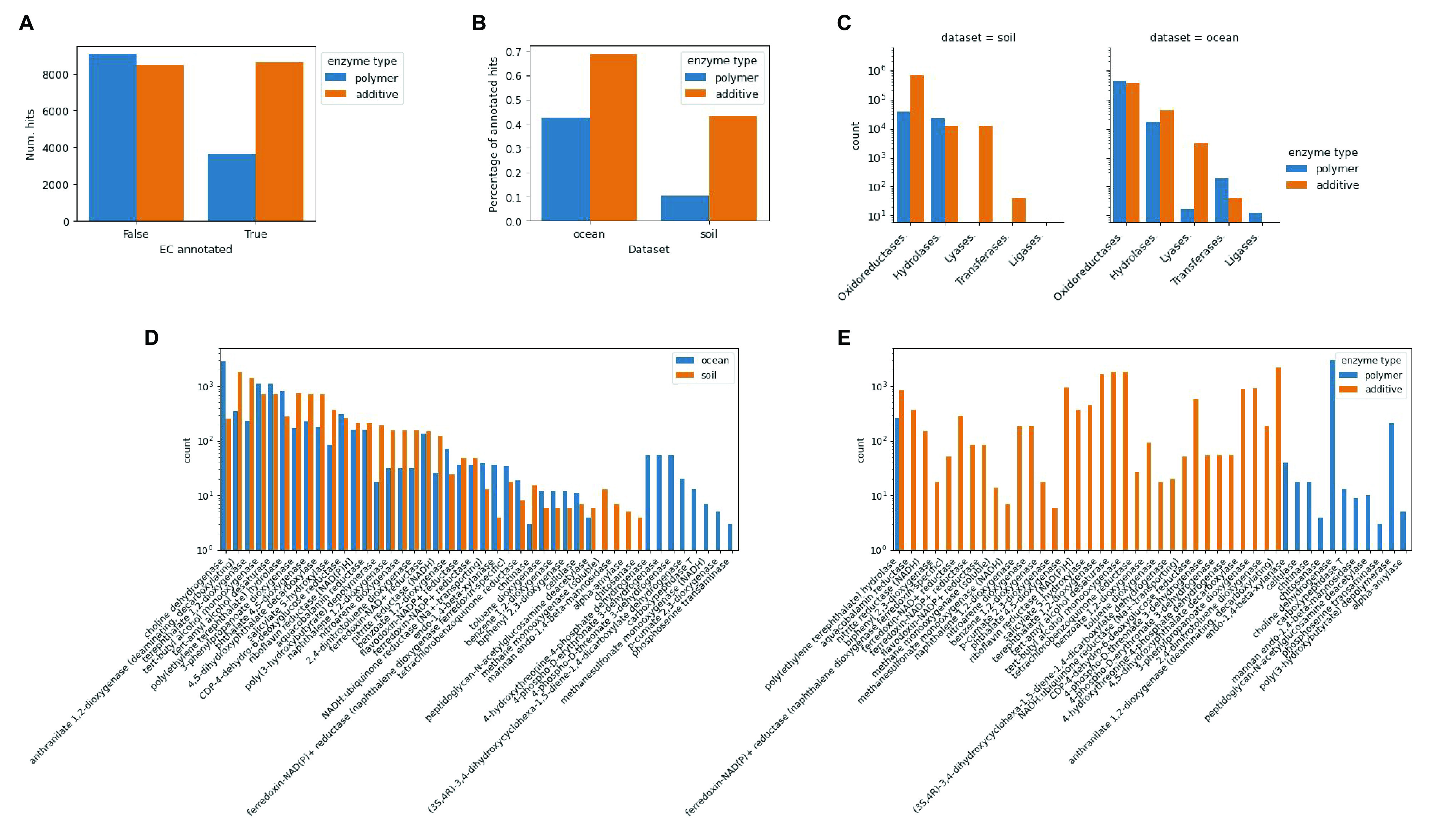

Supplement: FIG S4 [file mbio.02155-21-sf004.jpg]

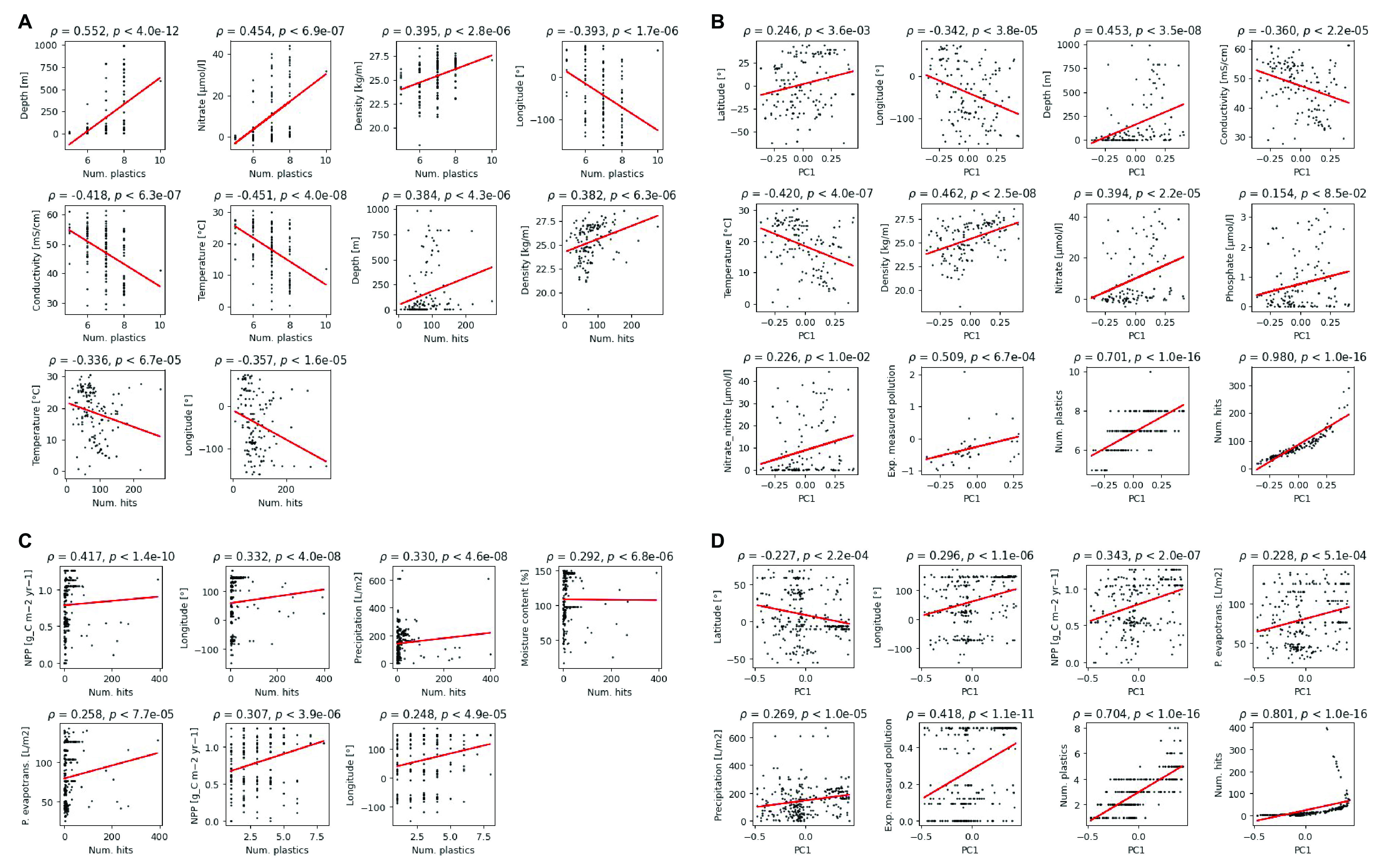

Supplement: FIG S5 [file mbio.02155-21-sf005.jpg]

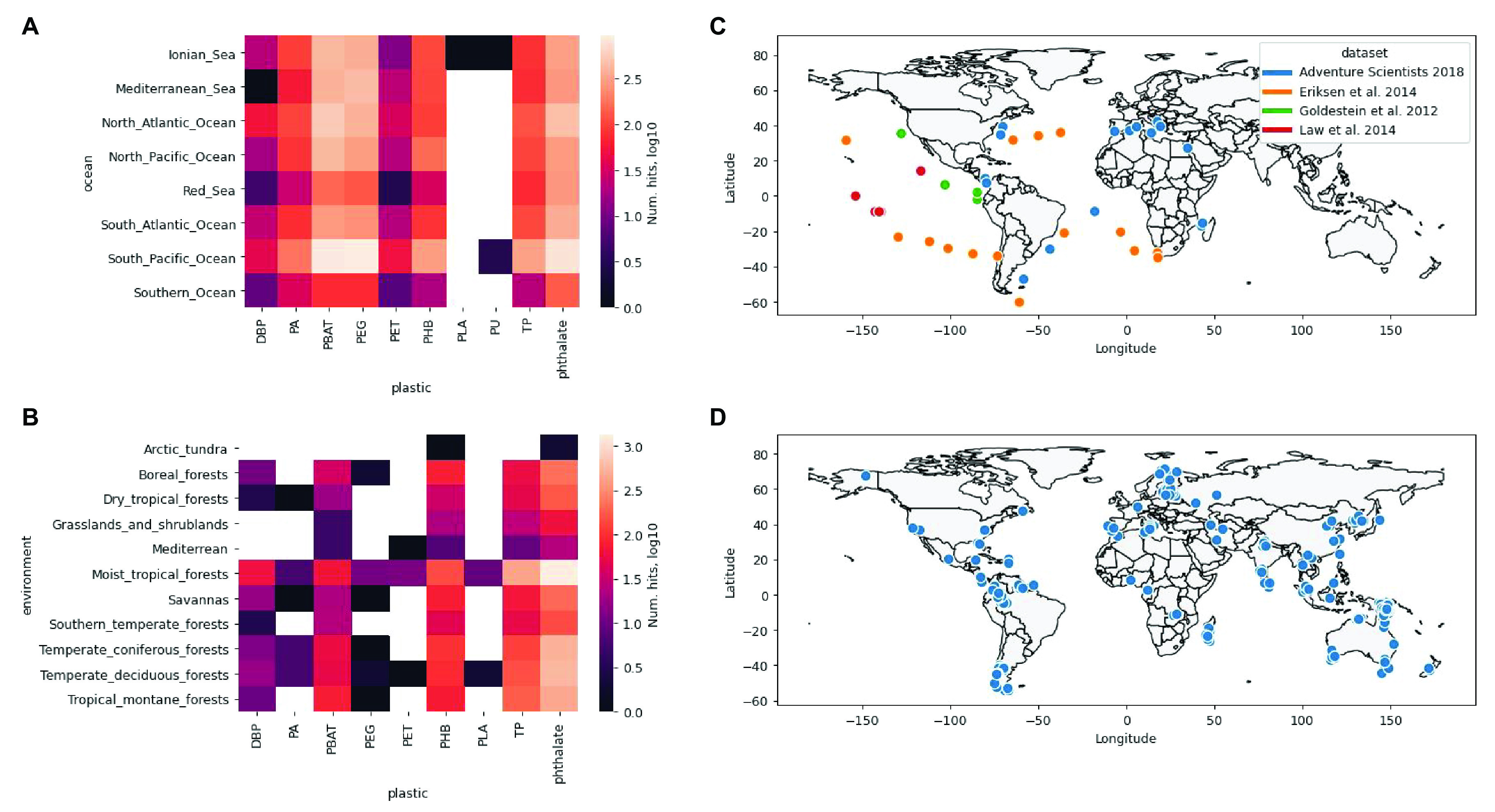

Supplement: FIG S6 [file mbio.02155-21-sf006.jpg]

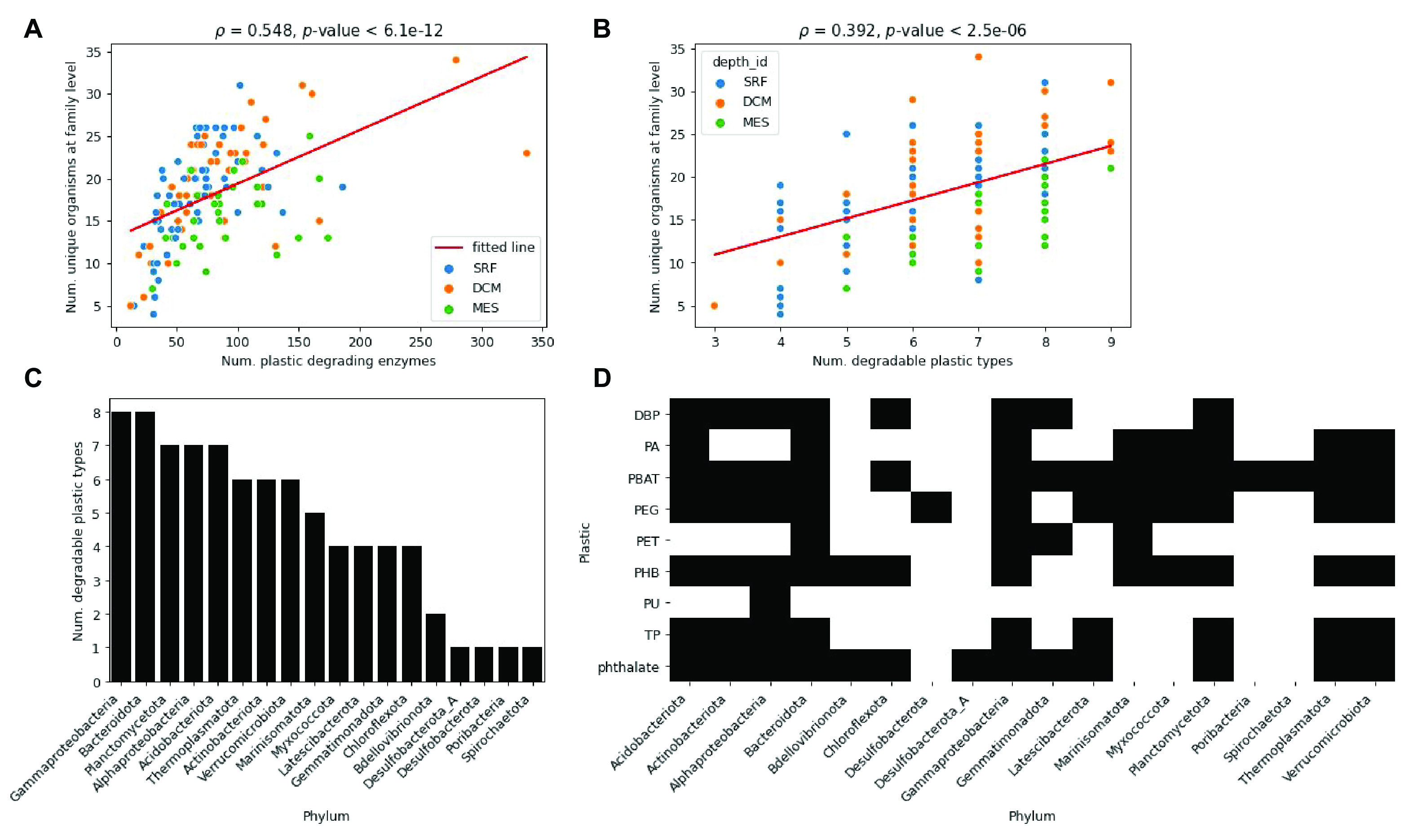

Supplement: FIG S7 [file mbio.02155-21-sf007.jpg]
